# The management of cognitive labour in same-gender couples

**DOI:** 10.1371/journal.pone.0287585

**Published:** 2023-07-13

**Authors:** Caitlan McLean, Connie Musolino, Alice Rose, Paul R. Ward

**Affiliations:** 1 Research Centre for Public Health, Equity and Human Flourishing, Torrens University Australia, Adelaide, Australia; 2 Stretton Health Equity, Stretton Institute, University of Adelaide, Adelaide, Australia; 3 Centre for Workplace Excellence, Justice and Society, University of South Australia, Adelaide, Australia; University of St Gallen: Universitat St Gallen, SWITZERLAND

## Abstract

**Objective:**

This study explored how cognitive labour as a form of unpaid, household labour is performed by people in same-gender couples.

**Background:**

Excessive performance of unpaid labour has been associated with several health impacts. Cognitive labour (anticipating needs, identifying options for meeting needs, making decisions and monitoring progress) is an underexamined dimension of unpaid labour which has centered on the experiences of heterosexual couples.

**Method:**

Dyadic and individual interviews were carried out to explore how cognitive labour was performed in same-gender couples between March and October 2021 using an inductive methodology. Adults who were in a same-gender couple, had lived with their partner for at least six months, were not living with children were recruited largely via social media.

**Results:**

Examining cognitive labour performance amongst same-gender couples revealed four key themes: 1) habitually fostered patterns of trust; 2) agency in redefining family; 3) barriers to cognitive harmony; and 4) facilitators to cognitive harmony. Findings regarding the relationships between themes are presented in a narrative model. Dyadic interviews allowed for deep, narratives relating to cognitive labour performance.

**Conclusions:**

The narrative model provides new conceptual understanding of how cognitive labour is performed outside of the heteronormative sphere. Couple’s adoption of a strengths-based frame to cognitive labour performance removed the opposition inherent in gender dichotomies. These findings support calls for research to incorporate social change to build and refine theory, including how queer and feminist movements have challenged gendered and heteronormative family and household roles.

## Introduction

Excessive unpaid labour has been linked to relationship breakdown, work-family conflict, poorer mental health outcomes, psychological strain associated with excessive role demand, and physical health implications including heightened night-time cortisol levels, reduced sleep and negative surgery outcomes [1–8]. Same gender relationships have seen a high degree of scholarly interest in the past decade, yet studies including lived experience, especially relating to cognitive labour, have received less empirical attention, with a large portion of research using quantitative methods [9, 10].

The present study seeks to inform theory and illuminate relational, social and temporal contexts relating to cognitive labour in same-gender couples [11–14]. *Same-gender couple* has been used in place of *same-sex couple* to maintain visibility and inclusivity of bisexual and transgender participants. Cognitive labour exists as a specific domain of household labour [15]. Cognitive labour has only recently been examined as an isolated form of work and further defined more recently which has aided in separating it from emotion work [16]. Historically, references to cognitive labour within household research have been vague, usually mentioned as a part of ‘women’s work’ with regards to planning meals, or as invisible work involving checking and policing of a family’s needs [17]. Its invisibility directly tied to the feminisation of tasks relagated to the private domestic sphere. Daniels [18] notes its importance as a labour form and the way *‘lip service’ is paid to the importance of this work’* but how outside of the market economy it is regarded as less important, considering how, for example, it is not included in the Gross Domestic Product. Cognitive labour encapsulates anticipating needs, identifying options for meeting needs, making decisions and monitoring progress within the household (Table 1). Examples of domains of cognitive labour include; food (deciding what food to buy and cook), logistics (maintaining family calendar), cleaning (coordinating unpaid and paid cleaning), and home and car maintenance (recognising needed repairs, finding repair professionals) [15]. Efforts by Dean, Churchill and Rupanner [19] to merge cognitive labour with emotional labour to form mental load are noted, although for the purposes of this paper we agree with Daminger [15] that cognitive labour should remain a separate entity of unpaid labour. Dean, Churchill and Rupanner contribute to the current cognitive labour research by expanding on Daminger’s work and concluded through a literature synthesis that the mental load (inclusive of cognitive labour) was boundaryless, invisible and enduring.

**Table 1:** Individual level recruitment data.

**Individual level data**	
Total participants	16
Mean age	29.8
**Gender**	
Man or male	4
Woman or female	10
Non-binary	2
**Sexual orientation**	
Gay or lesbian	13
Queer and asexual	1
Queer	1
Bisexual	1
**Work hours**	
Employed part time	1
Employed part time and studying	3
Employed full time	11
Not in paid employment	1

The extensive study of household labour, originating with an examination of economic theories and moving then to social constructionism, has continued to confirm the most consistent outcome of the research; women perform more housework than men, but this has traditionally been a finding relating to heterosexual couples [3]. Historically, feminists have noted that when ‘women’s work’ activities such as cognitive labour are ignored, the real work itself is not appreciated and therefore remains unacknowledged, and the impacts of this work on women’s physical and mental health undocumented. Drawing attention to these kinds of labour forms is a means to more deeply explore the way in which it is constructed [18]. There has been a renewed interest in the performance of cognitive labour and gender, with the Australian Institute of Family Studies highlighting recently that women in particular are calling for more attention on the performance of cognitive labour, especially in light of changes to household dynamics relating to the COVID-19 pandemic and subsequently exacerbated disproportionate performance of unpaid labour [20–22].

Studies which have specifically focused on cognitive labour have emerged in recent years [15, 19]. They have explored cognitive labour as a unique form of housework and have focused on heterosexual couples [15]. Cognitive labour has not yet been explored in isolation with same-gender couples [12, 15]. This paper examines how cognitive labour is negotiated and performed within same-gender couples, the reasoning behind its allocation and perception and how this may impact the individual and their relationship. Increasing our understanding of how cognitive labour is performed and experienced outside of the heteronormative dynamic (where it is disproportionately performed by women) will increase understanding, awareness and encourage changes to the way we perceive and accommodate cognitive labour in terms of paid work and family structure in relation to gender.

Additionally, this paper contributes to the limited qualitative research on the lived experiences of people in same-gender relationships in this field whilst employing the innovative method of dyadic interviews when examining cognitive labour, as a means of ‘*making the invisible more visible’* [19 p14].

### Gender, sex and sex characteristics

The conceptual distinction between sex (biological characteristics) and gender (masculinity, femininity and neutrality as social constructs) is not widely observed [23]. Some studies treat gender as a binary variable, whilst others treat gender as a unified concept (sex, gender and sexuality) [23]. There has not been a widespread distinction between a person’s gender and individual sex characteristics in health research [24, 25]. The biological paradigm foundationalism of gender presumes a person’s sex characteristics are an unchanging ‘base’ on which gender is socially constructed [26]. This study adopts a social constructionist approach to gender in an effort to move away from the traditional biological paradigm [23]. Gender is positioned as the category of importance, before other aspects of the person, such as biological characteristics [27]. This is because gender is an active process which is the outcome of social forces, and plays a significant role in the division of unpaid labour between couples and relationship structures [23].

### Household labour performance theory

Research on same-gender couples challenges theories relating to heterosexual couples’ division of labour and its reliance on gendered assumptions [28]. Current research suggests individuals in same-gender couples hold more egalitarian views and have more equitable performance of household labour than heterosexual couples, though this has not been addressed for cognitive labour [9, 29, 30]. Gendered housework theory shows same-gender couples are more likely to have similar housework preferences and both be employed outside of the home. There are also unique predictors present, such as work hours, time availability and income [28, 31, 32].

Although same-gender couples may not divide household labour equally, many defend their arrangements as fair [33, 34]. A large portion of research relating to same-gender couples and household labour revolves around lesbian families who are parenting. Findings from these studies show labour tends to be evenly divided between biological and non-biological mothers [29, 30, 35, 36]. There is a lack of understanding of cognitive labour for same-gender couples who don’t have children [9, 37]. Kelly and Huack [38] interviewed 30 queer couples in the US and explored the re/doing of gender through labour division and examined how couple’s negotiated domestic labour as a challenge to normative gender roles. While not addressing cognitive labour, they provide insight for broader labour practices. Couple’s accounts of unpaid labour were shaped by time availability and task preference but were also influenced more broadly by social contextual factors including employment and citizenship.

Existing studies addressing household labour are mostly survey-based, or use large national data sets, with few using qualitative interviews and even less using dyadic level data [39, 40]. Where surveys were employed, such as in studies completed by Kurdek [40] and Civettini [41], questions focused predominantly on the allocation of specific tasks and asked members of the couple to complete surveys individually. Goldberg [9] found a gap in the literature during their systematic review which highlighted the need for qualitative research in non-U.S contexts.

Goldberg [9] notes that current findings, when examined holistically show that labour division in same-gender couples may be more complex than the predominant ‘egalitarian framework’ that has saturated current literature. Future work should continue to theorise unique labour division among gender and sexual minorities [28].

This study addresses the limited body of qualitative data and delves into how same-gender couples navigate the dynamics associated with cognitive labour within an Australian population. It expands on conceptual understandings of unpaid labour and shows how couples challenging heteronormative assumptions can provide insights into the way we understand cognitive labour. Several explanations have been presented for how uneven unpaid labour distribution has come about [9, 37]. Below, broad research on the above theories, as well as gender theory, the egalitarian ethic and the heteronormative perspective are explored.

### Resource and time availability

Accounting for unequal division in performance of unpaid labour based on assumed differences in power, time availability and resources are all established theories [11, 42]. An individual’s relative time availability was amongst the most common factors associated with greater performance of physical household work. The studies did not differentiate between cognitive and other labour forms. Additionally, when it came to the role of gender, authors imposed masculine or feminine values to certain tasks, regardless of participant’s opinions and this could be seen as a reinforcement of heteronormativity as it pertains to household labour division.

Civettini [41] found, unlike patterns seen in heteronormative couples, financial power and net differences in earnings did not account for an imbalance in household labour performance in same-gender couples. Instead, differences in amounts of household labour performance within couples were linked to hours spent in paid employment, supporting the time availability theory, which has also been seen in other studies, and termed the time availability perspective [43–45]. This may suggest that resource and time availability constraints play a central role in same-gender couples division of household labour, but how that relates to the gender relations within the couple and household, needs further investigation.

### Task preference and skill

In a systematic review examining household labour among lesbian couples, Brewster [30] found existing theoretical frameworks remained relevant but recommended viewing labour performance in same-gender couples outside of the established heteronormative lens. Compared to heterosexual relationships, gender did not play as significant of a role in task division. Rather it was based on strengths, skill, and personal preference of the individual. Furthermore, the majority of participants did not report being dissatisfied in their relationship, valuing a just divide rather than an even one [29, 30].

Kurdek [40] employed surveys with American same-gender couples to examine the relative frequency of performance of housework tasks. They did not examine cognitive labour within any of the chosen tasks. When interest and skill relating to a task were examined, only interest accounted for the difference in amount of housework performed. Additionally, each person’s satisfaction with labour division affected overall relationship satisfaction and this is supported in other literature [30].

### Gender and egalitarian ethic

Gender and its influence on unpaid housework can be best explained through gender ideology and display [46–48]. Gender display theory sees the performance or absence of performance of unpaid labour as a medium to demonstrate gender and seek approval through the performance of gender roles [46]. At the core of gender display is the idea that gender categories exist as a result of behaviour (e.g. someone is masculine because they perform car maintenance) as opposed to role theory or gender ideology, where gender exists independently to the behaviour, and people customise their behaviours to suit these categories (someone does car maintenance because they are a masculine) [41–46]. Individuals are aware that by performing normative gender roles they can be provided with power, symbolic capital and agency [46, 49].

Empirical findings suggest same-gender couples are more attentive to issues of equality in their relationship when compared to heterosexual couples [50, 51]. While such an egalitarian ethic could be supposed to ‘de-gender’ housework, it has been argued inappropriate to construct any couple as being empty of gendered practices [52].

Expanding research to include same-gender couples allows for a broader understanding of how gender is ‘done’ and ‘redone’ through its performance, thereby seeking new paths and possible ways to reduce labour inequity [9, 37].

### Same-gender couples and cognitive labour as a specific domain of household labour

Daminger [15] completed semi-structured interviews with 35 heterosexual couples in an American population examining cognitive labour performance. This is the only study of its kind to focus on cognitive labour performance. It identified the dimensions of cognitive labour as anticipating needs, identifying options for filling them, making decisions, and monitoring progress. A literature synthesis was recently undertaken in response to increased interest regarding the impact labour forms such as cognitive labour may have on individual wellness. Women specifically have called for increased examination regarding the performance of cognitive labour and the mental load [15, 19]. Daminger provided a working definition of what comprises cognitive labour, but also demonstrated its gendered nature. Women in this study were found to do more of the cognitive labour work overall, especially planning and monitoring aspects. However, decision making, arguably the most power driven aspect of cognitive labour, was found to be more equal between men and women in heterosexual relationships [15]. The egalitarian nature of same-gender relationships suggests performance of cognitive labour may be more equitable than the findings from Daminger [19].

There remains an absence of research centered on how same-gender couples manage cognitive labour, how it is influenced and why [12]. Building on gender display theory and evidence from cognitive labour related studies with heterosexual and same-gender couples, this paper proposes that distinct couple dynamics, housework patterns, and emotion work affect cognitive labour performance.

## Materials and methods

This study seeks to understand perceptions of cognitive labour in same-gender couples. This includes understanding the function of cognitive labour performance and identifying barriers and facilitators to performing cognitive labour. Based on recommendations from Clarke [53] and Goldberg and Allen [28] this study does not centre an examination of sameness and difference. However, given limited literature specifically focusing on cognitive labour heteronormative research findings are discussed and drawn upon to explore existing theory. This study was approved by the Flinders University Human Research Ethics Committee (study no. 4106).

### Recruitment

Inclusion criteria for the study targeted participants who were in a same-gender, dyadic relationship, had been living with their partner for at least six months, did not live with any children and were over eighteen [23, 24]. This included same-gender cisgender, transgender and non-binary gender couples. There were no exclusion criteria relating to ethnicity, gender, sexual orientation or sex characteristics. Given established limitations to sample size and this being the first study to address this particular area of household labour with same-gender couples, exclusion criteria were kept minimal to facilitate higher recruitment. Those with children currently living with them were excluded given previous recommendations that parenting labour and care work should be studied separately from other forms of household labour as they draw on different theoretical arguments [2, 11].

Participants were recruited via posts on Facebook, Instagram and Reddit subgroups as well as university research advertisement services. Online, purposive sampling has been used in previous studies with LGBTQ+ populations and is heavily favoured given the strong online presence LGBTQ+ communities have fostered [39, 54–56]. The study was advertised as an ‘Exploration of the day to day lives of same-gender couples’ to avoid specific references to housework or labour load to avoid self-selection bias around this issue. A similar strategy was used by Daminger [15] and Pfeffer [39] to promote wider sampling.

LGBTQ+ people are known as hard-to-reach populations and can be difficult to recruit for study participation due to fear of stigmatisation and discrimination [57]. They can also be rendered invisible in ambiguous sampling frames. Participants were encouraged to pass on details of the study to anyone in their social circles as a means of snowball sampling to increase sample size. Basing recruitment solely online had the potential to exclude or limit reach to certain participants (e.g. people who do not use technology, those without access to an internet connection), however this was the most appropriate and viable means of recruitment given the nature of the study, especially given physical distancing requirements relating to the COVID-19 pandemic [58].

Involving relevant community partners has been found to greatly enhance recruitment success in studies with LGBTQ+ communities [57]. National community groups relating to LGBTQ+ communities and health who were deemed to have potential interest in the study were contacted with details of the study and an invitation to advertise and assist with recruitment.

### Methodological approach

Given the minimal established theory on cognitive labour and same-gender couples, a qualitative, inductive methodology using principles of constructivist grounded theory was justified [15, 59]. A constructivist approach assumes a relativist epistemology and acknowledges the multiple standpoints and realities of the researcher and the research participants [60]. A qualitative methodology allowed the exploration of participant stories without limiting or pre-defining their responses, and this was further embodied in the use of an iterative coding process in the data analysis phase [59, 61].

Individual level data are presented as the basic unit of analysis in most qualitative research, presenting with limitations in the exploration of phenomena that involves two sides, stories or people [62]. Individualistic data allows people to share information that might otherwise be held back, whereas dyadic level data encourages accountability and generation of new ideas and insights, such as a person’s partner’s perspective on the divide of household tasks [63]. This is the first study to use a combination of dyadic (joint interviews) and individual interviews to explore cognitive labour performance in same-gender couples. In their study exploring the difference between dyadic and individual interviews in the context of cancer, Morris [64] stressed the importance of offering couples choice between individual or joint interviews, but reiterated the wealth of knowledge a joint interview can give. The use of both forms of interview enabled deep content collection relating to the research objectives whilst fostering increased trustworthiness with participants [62, 65].

Both forms of interview focused on allowing participants to tell their own story of their experiences relating to cognitive labour. Dyadic interviews allowed for concepts and ideas to be discussed, questioned and reflected on in real time between the couples and the interviewer. This deepened responses whilst allowing for the capturing of emerging concepts and ideas, such as partner A being able to refute, support or question a comment made by partner B [62, 63]. Dyadic interviews allowed for a shared narrative to be developed whilst also highlighting relational characteristics, such as how partners may dominate or become subdued dependent on relational power differences [62].

The option for couples to choose either individual or dyadic interviews also promotes inclusion and prevents incidences where joint interviews may have posed a risk to participants or were not logistically possible. The aim was to develop a deep understanding of how participants constructed their understanding relating to the performance of cognitive labour within their relationship. A definition of cognitive labour was provided using real life examples such as meal preparation; where chopping of vegetables and washing up would be physical components, planning the meal, timing the meal, taking responsibility for cooking the meal and assessing which groceries needed to be bought ahead of time were given as examples of cognitive labour within the meal preparation task. During the interviews, participants were encouraged to expand on points they mentioned, allowing for ‘thick descriptions’ of experiences of cognitive labour to be elicited [66]. The interviewer promoted clarification of components of cognitive labour and perceived reasoning for how it was divided during the interview. Interview questions were framed around the elements of cognitive labour, and not focused on the individual people taking part in the interview in an effort to reduce risk of conflict. The interviewer adopted the role of being an outsider. Where contradictions occurred, couples were encouraged to revisit elements of their story as a means of ensuring interview understanding.

Similar to strategies used by Daminger [15], when respondents reported, for example, using a meal box subscription service in the context of talking about their daily routine, follow up questions were asked, such as; whose idea was it initially to use the service? How did it move from an idea to a part of your daily routine? This probing allowed for exploration of deeper cognitive labour processes underneath daily tasks.

An important aspect of the interviews, especially those which were dyadic was to ensure individual respondents were given opportunities to expand on what their partner had said. It was important this was achieved whilst also ensuring respondents did not feel pressured to respond or elaborate if they did not wish to.

The researcher conducting the interviews is a cis-gender, heterosexual person. It is crucial to acknowledge the epistemological and ethical implications of a heterosexual person researching LGBTQ+ people and the relationship between identity and knowledge production [67]. Self reflexivity was a key element of the research process and is central to sincere research [68, 69]. Throughout the research project the researcher conducting the interviews read a broad range of queer literature. They also kept a detailed journal throughout the recruitment, data collection and analysis process. Thoughts and reflections were then discussed during regular meetings with the other three members of the research team, one being in a same-gender relationship.

The knowledge a researcher produces is contingent on the researcher/researched relationship and how each person is positioned within overarching social structures [67, 69]. The interviewer was open about their sexuality during all interviews, thereby disclosing their outsider status to participants. The outsider status of the researcher was addressed as a means of encouraging participant’s to not leave out information that may have otherwise been deemed as irrelevant or trivial to an insider.

A large consideration within the study design was the risks that may occur during dyadic interviews, given joint interviews could lead to conflict [64]. Available evidence indicates LGBTQ+ people are as likely as non LGBTQ+ women to experience domestic violence [70–72]. There are several barriers to people in same-gender couples seeking help with instances of domestic violence, including; discrimination, insensitivity and lack of awareness from service providers and stigma [73]. Safeguarding measures were implemented as used in previous studies and as recommended by WHO [64]. This included provision of support service information for relationship counselling, individual counselling and domestic violence support as listed on the Information sheet. Participants were provided with direct contact details for not only the interviewer but also all three of the supervisors, one of whom is an experienced Lifeline Crisis Supporter, and these details were provided to both members of the dyad if participating in a joint interview.

### Study procedures

Interviews were scheduled online using Zoom Video Communications, a software used widely in previous studies which also met ethical expectations [74]. The use of video-enabled online interviews allowed the study to have a geographically dispersed sample otherwise not possible [74]. It also afforded a high degree of accessibility and flexibility to best accommodate participants and their personal schedules, especially in circumstances where the ability to attend in person would have been restricted due to factors such as COVID-19 social restrictions. Verbal consent to record audio and visual data was gained and data were then stored on a password protected computer that only the interviewer had access to. Participants were reminded that they could choose to withdraw from the study via email at any time until data reporting.

Audio and visual recording allowed for analysis of observations made during the interviews relating to pauses and body language as well as laughter, shoulder shrugging or nodding or shaking of the head. Consent was gained to contact couples again for further information if required.

## Data analysis

Interviews were transcribed using Descript, an automatic transcription software. Data for analysis consisted of the interview transcripts and observational notes written shortly after each interview. Analysis was then undertaken using NVivo software. The first author attached a memo to the beginning of each transcript that gave an overview of the interviewees and the context of the interview. This included participants’ responses to the Sexuality and Gender Indicators for Research [70], whether the interview was dyadic or individual and any potentially relevant demographic or contextual information about the interviewees.

Open coding was performed by the first author and focused on highlighting excerpts from each transcript and assigning a ‘code’ to them, also referred to as ‘microanalysis’ [75]. For each additional interview, the open coding process was repeated using an iterative process resulting in the eventual production of 565 initial codes. Initial codes were reviewed by the other three research team members. Given the minimal established theory, code labels remained close to the collected data as the analysis of raw data transitioned to forming focused codes. Verification with the research team through each stage of the data analysis process provided confirmation and verification of evidence for the developed codes. Developing initial codes with a high degree of specificity and rechecking concepts with the research team on a frequent basis strengthened the links made to focused and eventually theoretical codes, thereby increasing data integrity as the codes became more abstract. Each interview was analysed and coded using the same process regardless of individual or relationship attributes. Attributes were then considered once patterns started to emerge in the data later in the analysis phase. Initial interview questions were modified to reflect emerging codes as data analysis continued.

**Fig 1:**
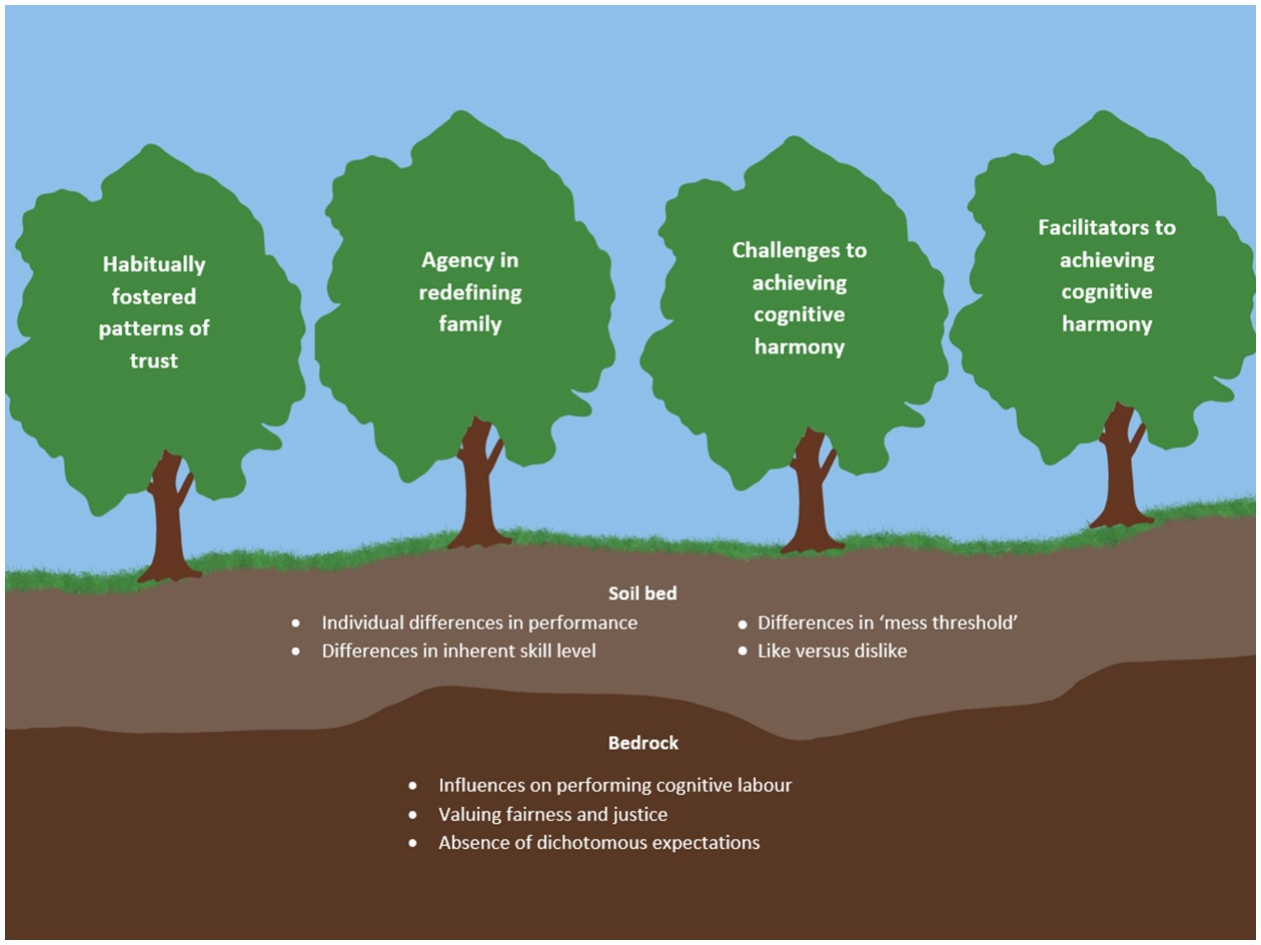
Tree model.

The research team met regularly to discuss the synthesis of codes and the development of initial interrelated concepts. Saturation was determined to have been met following the repetitive identification of existing codes and the absence of new ones during data analysis [76]. Once no new codes were identified, it was determined saturation had been achieved and led to a termination of data collection [77, 78]. Following the completion of data collection, the proposed theoretical codes were reviewed for how they related to one another and synthesised the categories derived from coding. Finally, the codes were reviewed for their boundary clarity, and quality of meaning, and an illustrative model grounded in the data was developed (Fig 1).

## Sample

Data for this study came from nine in-depth interviews, seven were dyadic and completed with both members of a couple and two were individual, meaning fourteen of the participants interviewed as a dyad and two participants interviewed independently without their partner (Table 1). Further information relating to the participants can be found in Table 2. Five people declined further participation in the study once the information and consent form had been provided. All interviews were conducted between March—October 2021, the researchers did not have existing relationships with any of the participants and no compensation was provided. One couple had recently relocated to another country at the time of the interview. No significant differences in themes between individual and joint interviews was noted during data analysis phase. Participants’ paid work varied widely by industry and included retail, hospitality, research, government planning and flight management. This sample reflects a significant variation in age of participants (range from 19 to 47) rather than ethnicity (Table 3). Participants in the study largely identified as being Anglo-Australian (n = 14) and two participants self-identified as Spanish (n = 1) and Argentinian (n = 1). Pseudonyms have been used throughout the study to ensure anonymity.

**Table 2:** Participant information.

Pseudonym	Age	Gender	Sexual orientation
Casey	45	Woman/female	Gay/lesbian
Claire	27	Woman/female	Lesbian/queer
James	34	Man/male	Gay
Jennifer	22	Woman/female	Lesbian
Jessie	27	Nonbinary	Queer
Kelly	32	Woman/female	Lesbian
Kim	26	Nonbinary	Asexual and Queer
Lachlan	32	Man/male	Gay
Laura	30	Woman/female	Lesbian
Lisa	26	Woman/female	Bisexual/pansexual
Melissa	28	Woman/female	Gay
Molly	19	Woman/female	Lesbian
Nick	30	Man/male	Gay
Sarah	24	Woman/female	Lesbian
Simon	28	Man/male	Gay
Taylor	47	Woman/female	Gay/lesbian

**Table 3:** Couple level recruitment data.

Couple level data	
Mean relationship length	3.8 years
Mean cohabitation length	3.3 years
Number of couples living in capital cities	7
Number of couples living in regional areas	1
Couple relocated overseas at time of interview	1

## Results

Through the conduction of dyadic and individual interviews, this paper shows how same-gender couples formulate different roles based on their past experiences. Analysis of the data generated a unique set of relationships between six themes that resembles a natural ecosystem (Fig 1) which shapes how cognitive labour is performed in participant relationships. Each person enters the relationship with their own ideas surrounding cognitive labour, represented in the model by the ‘Bedrock’: individual contextual factors of each person’s past experience and perceptions. In addition, the data featured an interplay between each person’s individual contextual factors within the domestic setting; the ‘Soil’ layer. The intermingling foundations of bedrock and soil provided the contextual basis–an ecosystem—from which four types of trees grow to shape the performance of cognitive labour of participants’ same-gender relationships: ‘Habitually forming patterns of trust’ (Tree One), ‘Agency in redefining family’ (Tree Two), ‘Barriers to achieving cognitive harmony’ (Tree Three), and ‘Facilitators to achieving cognitive harmony’ (Tree Four).

The four trees were present in each couple’s relationship, growing uniquely dependent on their circumstances and representative of different aspects of cognitive labour performance which exist alongside one another. While the four themes are discrete, trees were chosen to represent the dynamism of their presence in the ecosystem. For example, in some relationships, one person may give primacy to trust when navigating cognitive labour demands (Tree One), while agency (Tree Two) a highly salient for their partner. Additionally, the trees allow for a dynamism identified in the data, as though intermingling branches in a forest. While the data indicate some variation the level and nature of relationships between the themes, the ecosystem model allows for flexibility while remaining representative of the sample.

### Bedrock: Context of the person and outer influences

*“And I think when you have to go through that hard road of getting clarity on your identity*, *in a world*, *that tells you*, *you shouldn’t be those things that you should fit neatly into all these other little spaces that you get an inner strength in a way that you can’t get*, *unless you have to walk through fire in that regard*.*”*

(Casey)

In traditional housework studies, there is a large focus on how ‘genderisation’ of housework impacts performance, ultimately determining who does what, and why they feel they should do certain tasks. Participant’s stories pointed to other influential concepts; a resistance to the norms they saw in their parent’s performance of unpaid labour, the absence of a dichotomous relationship dynamic impacting their formulation of ideas and roles in a relationship, and the underlying importance of fairness and justice.

These influences lead to the formulation of differences between each person in how cognitive labour was performed and valued. All participants reported growing up with parents who were in a heterosexual relationship or with a single parent. Several participants shared how their childhood home had influenced their own needs relating to cognitive and household labour performance in their current relationships, whether that be in molding their personal preferences for when tasks should be completed, or choosing to take on a higher cognitive load:

“*It’s something that I was doing beforehand for my mum*. *Because she’s a single mum that works a lot*. *So I was already doing her food shopping and that kind of thing*… *I think that I take on a little bit more of that role in general throughout the whole relationship*, *which is again*, *something that I’ve been doing a lot of my life with single parents and I have younger siblings*.*”*

(Molly)

Sarah suggested that differences between how her and her brother perform cognitive labour was at least partially shaped by their socialization growing up:

“*My brother just kind of likes to wait for things to happen for him before he will try and do it for himself*. *I find it interesting that we both grew up exactly the same and yet*, *I am a little more mindful about your (Claire) feelings and doing my part in the relationship*. *But he*, *is a bit more relaxed about it and I suppose it might come down to that whole societal thing where men grow up to be more entitled due to multiple factors.”*

(Sarah)

Couples identified that in the absence of gendered heteronormative dichotomous relationship expectations, they were better able to balance the cognitive load without projected constraints they had seen in relationships between men and women. Lisa stated “*We’re living in a society where it’s a female’s natural role and need to nurture and take care of*, *whereas the male’s natural nature*, *to protect and provide and all that kind of stuff*. *But*, *we’re both women*. *laughs* So yeah, *so we kind of share the load because we don’t really adhere to one or the other*, *but I think we both have aspects*, *just naturally within our own personalities*.*”* This consequently led to couple’s having to refute people’s attempts to force this dichotomy on their relationship: “*We still get people that*, *every now and again*, *it doesn’t happen so often nowadays*, *but we’re always asked who’s the man in the relationship*? *People just love to have that- the box.”*

An existing knowledge of gendered inequalities in household performance was reflected in couple’s relationships and the need to adopt a strengths-based approach to labour division:

“*I think when we*, *because we’re aware of the data about*, *you know*, *women do way more and stuff*. *And when we moved in together*, *the first time I think we did kind of have a conversation about it and really just kind of came to this strengths-based approach*.*”*

(Kim)

Queerness was also seen as an alternative to the dichotomous relationship of household labour performance and gender norms and allowed them to be more reflective of gender inequalities:

*“I definitely think there is some relationship between queerness between*, *transness and sort of having the*, *self-awareness and the flexibility to be like*, *there’s no one sort of assigned role that I have to get into*.*”*

Couples did not report an equal division of cognitive labour. Rather, it was a perception of fairness, and that the division should be fair and equitable, not even. Participants shared how valuing fairness and justice in the wider world and seeing unequal division in their childhood homes influenced their current relationship:

*“And then she* (participant’s mother) *would remake the beds*. *And it’s like*, *all of this stuff*, *obviously I only picked up on it in my early teens*. *But thinking about that from a gendered point of view*, *it was definitely like*, *early stages of me being like “well hang on*, this is not fair” and being a teenager I was like “this is f***ed.*”*

(Melissa)

“*But I remember them (Jessie) saying*, *look*, *things don’t have to be perfectly equal all the time*. *That’s not always what’s best or what’s fair*. *It’s about what we can do*.*”*

*(Kim)*

### Soil: The interaction of each person’s context and cognitive labour performance styles

*“I don’t think that we’ve necessarily discussed it in depth*, *but I think that we have different ideas of what that (cognitive labour) is*. *I’ve always been much more fly by the seat of my pants type*. *Whereas I married a planner*, *through and through*. *I’m more disorganised and chaotic*, *whereas he is more organised and structured*.*”*

(Simon)

Previous experiences within a person’s bedrock foregrounded the negotiation and performance of cognitive labor in their relationship, facilitating the creation of unique patterns in labour performance. Additionally, the combination of each person’s foundations contributed to how people formulate their cognitive labour routine for their particular dynamic. Contextual factors may weave in and out of the interpersonal relationship (including factors such as paid employment). This study found the way same-gender couple’s actively navigated and integrated their own differences led to four different elements of cognitive labour performance within each couple. Some participants described performing more cognitive labour than their partner as a means of keeping control, other participants spoke about preferring or being inherently better than their partner at certain cognitive labour tasks. The idea of people having a different cognitive load or mess threshold also became apparent:

*“I think Kim likes to tick things off*, *but in terms of remembering*, *it just seems to be a little easier for me to remember*, *to do things by a certain day*, *or remember to write down*, *to do it by a certain day*.*”*

(Jessie)

Performing more cognitive labour to keep control and reduce anxiety was a mechanism shared by several women across the interviews. Lisa explained that she focused on long term planning and organising because of her anxiety. Similarly, Casey and Taylor explained how because finances caused Taylor stress, Casey performed the majority of financial management:

*“I’ve been on the bones of my bum for many years as well in extreme poverty*. *But*, *I’m not afraid of having nothing*. *Again*. *I know that I’ll always be alright*. *It doesn’t stress me out the same way it does Taylor*. *So*, *because it stresses her out so much*. *I actually manage our money*.*”*

(Casey)

Lachlan described the distribution of labour with his partner, Nick, to ‘trading on comparative advantages.’ When asked about how cognitive labour is performed in their relationship, Kim recalled how they divided tasks by strengths with the partner Jessie, and what each person didn’t hate, with other couples voicing similar reasoning:

*“It makes it more bearable if you get to do the things that you like or slightly prefer to do than you know*, *just being stuck with the task that you just like*, *argh*, *here we go again*.*”*

(Laura)

Participants displayed varying tolerances for the intensity of cognitive labour they could perform, which

influenced how they chose to perform and divide cognitive labour tasks.

***“****We definitely think very differently*. *She probably isn’t making as much of a mental list*. *She likes to tell me all the time that she doesn’t have thoughts*, *which is really funny*. *I find it really funny*. *I’m like*, *I just overthink everything*. *And she’s just like*, *“I don’t have thoughts”*, *like*, *what do you mean*?*”*

(Molly)

This also involved a comparison to their partner’s style and threshold:

***“****I think with like the mental*, *one of the differences is*, *I’m not sure I would necessarily get to an overload state or an overflow or whatever*. *Whereas I think you get to that overflow and then you’re like*, *all right*, *well*, *I’m doing all these things*, *you can do it*.*”*

(Simon)

Couples linked their performance patterns to originating from a foundation of trust, attuned equity, communication and maintained independence within their relationship. For some couples, factors within the bedrock and soil components of the model had not been previously discussed. The interview was the first time they had openly discussed influences on cognitive labour division as a broader concept.

### Tree one: Habitually fostered patterns of trust

*“We both have made a really important point to say before this escalates*, *before we get pissed off and into an argument*, *can we communicate with the other*, *Hey*, *I feel like I’m taking on a bit more at the moment*.*”*

(Lisa)

Couples shared the fundamental ways they fostered trust in their relationship. An underlying assumption that each person trusted the other and held no expectations relating to the other’s performance meant cognitive labour could be tailored to suit individual need. Jonathan [79] describes this fairness and paying equal attention to needs as ‘attuned equality.’ Open and clear communication was attributed as largely responsible for how couple’s divided cognitive labour successfully. Couples linked their thoughts around cognitive labour to an initial expectation of why they chose to live together:

*“I think it’s something that’s really important in our household*, *is just that open*, *clear communication before things get blown up and out of proportion because neither one of us want to be fighting*. *We’re not*, *I’m not here to fight her*.*”*

(Kelly)

A mutual understanding of the importance of individual wellbeing over expectations held around performing cognitive labour was also established. Because of a high level of trust each person’s performance of cognitive labour, couples ensured a focus on individual wellbeing, rather than equal division. This was talked about by couple’s in relation to expectations:

*“It’s the expectation that*, *you know*, *I’m going to have a full day off or Lisa’s going to have a full day off*. *And*, *and that’s fine*. *At the end of the day*, *we have to make sure that we both have enough energy to be ourselves*. *I’d rather that she did that than killed herself*, *trying to get the house clean or something*.*’*

*(Lisa)*

Cognitive labour was established not as a static ‘chore’ performed by one person, but as a set of tasks which could be re-allocated dependent on the needs of the individual and over the life course. This included not just mental or physical health and fluctuating paid work demands, but also study requirements:

*“When I was towards the end (of an honors thesis)*. *I was like*, *I can’t do anything else*. *I have no time and no energy for anything else*. *24/7*, *I’m thinking about this or I’m actively working on it*. *And*, *all I can do is just*, *have a shower and eat and*, *I need help*. *So I was like*, *Kim*, *I’m really sorry*, *I can’t take on this responsibility right now*. *And can you help me*?*”*

(Jessie)

A change in division of cognitive labour also presented a temporal element. Couples discussed how expectations and needs relating to cognitive labour performance were likely to change over the life course:

*“I think that as*, *as we grow together and things change that we’re going to be good at redefining what needs to be done and in whatever order and our communication is so good and so open that the picture that we have looks ideal for right now and when things start to change or*, *you know*, *whatever life might throw at us*.*”*

(Kelly)

In contrast, traditional, dichotomous gender roles relating to housework division are often viewed as static and limit this flexibility [45]. Amongst couples, there was a shared expectation that each person would do what they could on any given day, fostering and reinforcing an underlying trust:

*“It’s the ebb and flow of the need of the household*, *the ebb and the flow of the person’s health*, *their emotional wellbeing*, *the seasons*, *like all those things are going to affect how much each person works*.*”*

(James)

*“In terms of what’s fair*, *it’s not necessarily splitting everything 50/50*. *But*, *I think we both feel the other person will do what they can*.*”*

(Jessie)

### Tree two: Agency in redefining family or household

*“I’m just always thinking up here ‘how could this house be gayer*?*’*”

*(James)*

Participants spoke about the power they held in redefining their household. This included how cognitive labour was performed and the strength that came from a freedom from broader socio-cultural expectations around who should be responsible for cognitive labour. Couples spoke of a freedom in being able to ‘make their own rules’ for how cognitive labour was performed outside of heteronormative constructs:

*“That was one of the first things that I was like*, *“this is an awesome part of being queer and creating your house*.*” And so for me*, *I’ve just thought*, *it doesn’t have to be*, *there’s a person who always does these things and a person who does these things and those lists better be the same length*.*”*

(James)

For Simon and James, this did not necessarily mean an even performance of cognitive labour. They both agreed James performed the majority of tasks (e.g., responsible for finances, keeping track and scheduling irregular house tasks such as winter preparedness, food planning, delegating of tasks within the home). They also agreed this worked within their relationship and remained dynamic based on what each person wanted:

*“I think part of my philosophy of household division of labor sharing the emotional and cognitive load*, *goes back to my perspective on like what a queer household is*. *And like a 50/50 division of labor*, *I don’t know*, *we can*, *we can create whatever we want the house to be*.*”*

(Simon)

Couples acknowledged the interplay of their division of cognitive labour was centred on respect for one another and not on an existing ideology around gender or labour division:

*“And so we don’t take any of that for granted*. *We don’t follow any gender stereotypes*, *any*. *We just do what we want to do*. *And we look after each other and we make sure we both feel respected*. *And it’s very*, *very simple*…*We’re both here because we want to be*, *we don’t have to be*. *We’re here because we love each other and we want to be*, *you know*, *we want to live life and be happy*, *you know*?*”*

(Casey)

The traditional breadwinner-homemaker ideology was challenged by the participants. Couples highlighted how the experience of friends and acquaintances in straight relationships, where uneven cognitive load had created conflict, reinforced what they valued in their own relationship:

*“Predominantly our friend’s are straight and it’s typically*, *you know*, *women talk about the gender division of labor in their relationships without talking about it in that way that’s what they’re talking about*, *how they go to work*. *And then when they get home from work at six o’clock*, *six thirty and they’ve still got to look after the kids and make dinner and all that sort of stuff*. *I guess it reminds me the impact that has on me is that that’s just not the life I want*.*”*

(Casey)

As couples shared their stories it became apparent that paid work was also not static, and the demands of employment did not permanently dictate who was responsible for cognitive labour. Couples intentionally negotiated how cognitive labour was completed in their relationship whilst also modifying its performance depending on their circumstances. Renegotiation of cognitive labour occurred dependent on factors including seasonal paid work changes, study load and wellbeing. This was a core element of how couples embodied the idea of a ‘queer household’ to allow agency and space to challenge the gendered dichotomy of cognitive and broader unpaid labour.

### Tree three: Challenges to cognitive harmony

*“I mean*, *you’re probably talking to other people who do have a lot of imbalance*, *so it’s not like*, *this queer-utopia*?”

*(Kim)*

Every couple shared ways in which they achieved a homeostasis of cognitive load, or cognitive harmony, and how this was both inhibited and cultivated at various times. Couples explored feeling guilt and responsibility to perform cognitive labour, their performance going unnoticed for prolonged periods of time, cognitive labour avoidance, being unaware of each other’s expectations relating to cognitive labour performance and avoiding talking about cognitive labour. These challenges formed tensions in the relationship, leading to stress and interpersonal conflict:

***“****I think that*, *in the moment I’m very vocal about my expectations*. *Whereas*, *and sometimes I can come across brash or blunt*, *whereas I think that you wait until things are kind of bothering you to voice those expectations*.*”*

(Simon)

The presence of a delegator and a delegatee was a common occurrence. The delegator performed a higher amount of cognitive labour tasks, given delegating is a central aspect of ‘household management.’ Whereas the delegatee was in the position of being given instruction or direction for performance:

*“Taylor*: *It’s fine*. *I mean if Casey asked me to do something I’m happy to do it*.

*Casey*: *Yeah*. *It annoys me sometimes*.

*Taylor*: *Yep*.

*Casey*: *Yeah*, *it*, *it really irritates me sometimes*, *but it’s really not a big thing to get irritated about*. *You know*, *like it’s just one of those things and yeah*. *We joke about it*, *I’ll*, *you know*, *prompt Taylor*, *or mention it and say*, *Hey*, *could you do that while I do this*? *And she’ll do it*.*”*

This further supported the need for a deep fostering of trust and communication to manage dynamic cognitive labour needs. In contrast, Nick was identified as the main performer of cognitive labour within his relationship with Lachlan. Although he reported being happy to be the one delegating, and likewise, his partner Lachlan agreed he was happy to be delegated to.

Dyadic interviews offered a space for shared reflection and correction [80]. At their core, dyadic interviews allowed couples to hold each other accountable whilst framing their narrative to an outsider. Couples course corrected instantaneously, highlighting how cognitive labour is invisible during day to day performance, especially to those not performing it, as first seen by Daminger [15]:

*(Couple were asked how they view cognitive labour split in relationship*)

*“Simon*: *70/30*? *80/20*?

*James*: *Who’s who*?

*Simon*: *I’m 80*, *you’re 20*.

*both laugh*

*James*: *You think so*?

*Simon*: *No*, *I’m teasing*. It’s like 80 *points to James* 20 *points to self* or 70, *30*.

*James*: *That’s more in balance than I think it is*.

Across all interviews it was clear when cognitive labour tasks were disliked by a person they were more unlikely to be conscious of the task being performed or of the task’s necessity. Phrases such as “that’s not even on my radar,” or “I don’t have to think about that because I know they will do it” were common:

*“I’ve only cleaned the bathroom once or twice since we’ve lived here*, *because I know that that goes on your list without me asking for it*. *You take initiative for that*. *So that just*, *there’s some things like that*, *that don’t ever go on my list because I know you notice them before I think of like prepping for it*.*”*

(James)

### Tree four: Facilitators to achieving cognitive harmony

*“Honestly*, *we’re actually—and it’s something we’ve commented on before*, *we are pretty good at recognising or taking on board when the other is struggling and picking up the slack*.*”*

(Melissa)

Couples established rules and systems that focused on their strengths and needs, incorporated strategies to reduce cognitive labour load, jointly managed cognitive labor whilst still centering individual performance and accepted differences in each other’s expectations as a means of facilitating cognitive harmony. Where a couple identified that cognitive labour was less than manageable, instead of one person increasing their labour, the couples worked together to integrate a range of strategies to reduce cognitive labour overall.

The use of google calendar, electronic shopping lists, online food ordering, autopayments or direct debiting for bills, scheduling robotic vacuums and use of meal planning and dinner subscription boxes were among strategies identified by the couples. A centering of individual responsibility also grew from each partner having an inherent trust that their partner would perform cognitive labour, if they said they would:

*“Yeah*. *And it works*. *I’m not like*, *do it right now*. *But like*, *with the joke*, *how people are always like*, *you know*, *‘if your partner doesn’t do it*, *you’re like*, *oh*, *I’ll do it myself*.*’ No*. *I know that she’s going to do it*. *She just needs to*, *you know*, *she’ll do it when she finds the mental strength to get to it*.*”*

(Laura)

Couples also referenced making cognitive labour practices intentionally flexible for when paid work or study commitments changed and therefore their labour load at home also needed to change. One couple linked individual performance with their shared goal of maximising time spent together, given seasonal work commitments required living apart for weeks at a time:

*“Especially*, *you know*, *the fact that I do travel*, *we’re very conscious of making the most of those times when I’m home… But if I have the night off and I’ve got no plans or anything*, *I’ll do a big*, *big clean*. *Just to get it done and out the way*, *but nothing else on my case*. *Yeah*, *that way we can spend more time together during the week*.*”*

(Kelly)

## Discussion

This study established a framework for understanding how cognitive labour is performed by same-gender couples. The formation of four distinct concepts (or trees) relating to cognitive labour performance were identified; habitually fostered patterns of trust, facilitators to cognitive harmony, challenges to cognitive harmony, and agency in redefining the household. The exploration of how cognitive labour is performed by same-gender couples contributes to the call to change how we explore and research unpaid labour more broadly. This study highlights that while theories such as resource and time use help to explain the external constraints and resources available to the couples when managing cognitive labour division, for the same-gender couples in this study, they are not the driving force. Within the tree model (Fig 1) we locate contextual factors such as financial resources and time availability due to work or study load in the *bedrock*. Time availability and resources did influence how couples allocated the division of unpaid labour, but it is in the *soil* that we see how the same-gender couples negotiated these factors moves beyond the taken for granted heteronormative concepts which underpin much of the theory on labour division. The findings demonstrate how an interplay of factors contribute to same-gender couple dynamics concerning cognitive labour.

Whilst participants seemed more aware of their cognitive labour decision making than in the study by Daminger [15], these active roles may not be articulated between couples. Centering cognitive labour not as a static labour set automatically assumed, but as something that is re-evaluated based on each couple’s unique circumstances was evident. Gender ideology and gender display relating to housework assume that heteronormative gender roles are internalised uniformly across groups of individuals [81]. This supports findings from Goldberg [9], who acknowledged classification systems dividing labour forms as inherently masculine or feminine only serve to perpetuate gendered meanings associated with housework.

In contrast to the work done by Daminger [15] cognitive labour was dynamic between each person dependent on individual need, rather than being habituated. This challenges the assumption that doing gender in housework is a static, concrete process enacted consistently across all couples. We do not dispute that housework, specifically cognitive labour is informed by gendered social constructions. What is evident is that same-gender couples were redoing gender, and cognitive labour performance was not tied to existing assumptions.

In comparison to recent work on mental load by Dean, Churchill and Rupanner [19], cognitive labour was examined and discussed by participants separate to emotion work and was instead in line with Daminger’s components. It is anticipated that if parenting related cognitive labour was to be examined there may be a stronger emotional component than seen in this sample. This present study adds to the growing body of cognitive labour research and affirms it invisible, boundaryless and enduring nature. It also encourages a revision in the way household labour is managed as findings showed how couples navigated labour division outside of purely examining time use or through quantitative measures. Examining cognitive labour requires a more in-depth approach and inclusion of specific cognitive labour based lines of questioning as well as physical labour as a minimum.

Investigations of same-gender couples’ housework shows higher rates of equality in performance when compared to heterosexual couples [9, 29]. There has been a link made to a decreased importance of gender display and a shift towards equality and personal preference. This study found same-gender couples do still consider time availability and relative economic resources, but not in the way that has been widely explained in existing research. Instead, same-gender couples consciously acknowledged these factors and examined them closely.

As the theoretical base for housework performance has grown, our understanding of household labour has become more complicated and nuanced [82]. This model supports the assertion that as we find less evidence over time that points to traditional heteronormative gender ideology as motivating cognitive labour performance, a change in gendered time use as well as the gender ideologies supporting it more widely may result [36].

Contrasting themselves with their familial upbringings and heterosexual friends, same-gender couples centered the self and a knowledge of the needs and strengths of their partner. Couple’s adoption of a strengths-based frame to cognitive labour performance removed the opposition inherent in gender dichotomies. This allowed for couples to feel in control of the performance of cognitive labour, supporting the call for research to use social change as an opportunity to build and refine theory [82].

Historically, disagreements relating to housework have been associated with increased depression [83], relationship dissatisfaction and stress [84]. Additionally, the perception of fairness of distribution of household responsibilities accounts for both individual wellbeing and relationship satisfaction, whilst the opposite perception can be responsible for increased conflict [6]. Cognitive labour load is not necessarily split evenly amongst people in same-gender couples, despite couple’s voicing egalitarian values and a sense of fairness. Cognitive labour division can be influenced instead through foundational relationship factors, task specialisation and task preference. These findings affirm equal labour division is not a realistic or even a desirable goal for housework amongst modern families [6, 81]. Examining cognitive labour performance in same-gender couples has provided alternatives for considering cognitive labour performance.

Strazdins et al. [85] argues time is a resource individuals must have in order to achieve good health. Combined with the fact that paid work structures are changing and blurring the line between work and home through flexible work models, it has become increasingly difficult to strictly define time used for paid versus unpaid work [81]. Where same-gender couples identified an imbalance in their performance of cognitive labour, and arguably a time deficit, they did not solely rely on their partner to compensate their performance. There was no assumed responsibility for cognitive labour, rather responsibility was chosen by one or both people and redefined dependent on individual needs. This reflects the need to challenge existing assumptions relating to housework and time use. Research suggests dual earner couples are more likely to have the means to ‘buy out’ or afford more costly forms of automation and assistive technology, thereby reducing labour load. This sample consisted of all dual earner couples, bar one, and therefore considerations need to be made for couples in different social classes, and different cultural contexts [86].

Popay, Rogers and Williams [87] provide a comprehensive rationale for the assessment of qualitative health research. A strength of this study is how it meets the primary marker of lay accounts and the privileging of subjective meaning. The data analysis process ensured research reporting not only privileged but centered the voices of the participants with strong links evident between the original data and the themes generated.

There has been a resurgence of dyadic data in contemporary literature [62–64]. It is a strong methodology given its ability to deepen content relating to couples as well as increasing trustworthiness [62]. Themes and content were consistently found across both interview types, however it should be reiterated that only two individual interviews occurred. The presence of two people during the dyadic interviews the subjects discussed were able to be explored much deeper. It is important to acknowledge couples may have modified their responses during dyadic interviews due to the presence of their partner. It has also been suggested that joint interviews may cause antagonisms between couples to surface. However, this can also be noted as a limitation for individual interviews, given couples may want to know what was said about each other [64, 88]. During individual and joint interviews, questions were framed in an effort to avoid antagonising by focusing on the practice of cognitive labour, rather than the individual performance of it by either person in the dyad. The authors believe this approach also helped to minimise risks of conflict elevation which was further aided by the sample having egalitarian values.

This was a relatively small-scale qualitative study. A large sample was not anticipated, especially working with a hard-to-reach population group and given a primary, qualitative methodology does not allow for use of large national data sets and data us limited given the relatively small amount of people within this population group [89]. However, results do provide a good basis for exploring new understandings and experiences of unpaid labour within the home that more fully encompasses contemporary families. The sample is not a statistical reflection, it is instead a reflection of the wealth of information the sample gives and is in line with theoretical, purposeful sampling as recommended by Popay [87, 90]. Although some organisations provided feedback regarding recruitment and study design, this did not prove to be a successful recruitment pathway in this instance.

People from this study mostly self-identified as white, were well educated with relatively secure employment. Future studies should explore differences related to social class, ethnicity, income level and level of education. The sample consisted predominantly of couples where both members were willing to speak, which likely resulted in a cohort that was not only more open about cognitive labour performance, but also had lower occurrences of conflict relating to housework and potentially a more egalitarian ethic than the broader population.

Couples were in comparatively short relationships, with a mean relationship length of 3.8 years. Differences in power and therefore potentially labour division are likely to occur over the life course and relationship length increases, particularly if couples were to have children [91]. Timing and dynamics of a first sexual-minority or heterosexual relationship would likely impact on subsequent relationship dynamics and this would include how cognitive labour may be divided within said relationship [28]. While heterosexual couples also espouse egalitarian values regarding the division of unpaid labour, evidence suggests in practice gender underpins and constrains the division of labour in practice [4–6]. Future research could look at the sustainability of using fairness and serving each other’s needs as a reference point for cognitive labour distribution and how performance may differ for same gender couples with children.

Additionally, there was a large age range between couples with differences between couple’s influences on cognitive labour evident depending on age. For example, the oldest couple in the group linked past relationship experiences to their values and perceptions of cognitive labour, where the youngest couple relied on influences from the parents more so than previous relationships.

## Conclusion

Like Kelly and Huack [38], this research advocates for the reviewing of wider household divisions of labour with a “queer eye”. This present study of how same-gender couples navigate cognitive labour has provided further evidence for the need to shift away from the ‘traditional’ study of housework labour. A model was developed which shows how a person’s contextual factors interact to form four key themes for cognitive labour performance: having agency in redefining household, habitually fostered patterns of trust and challenges and facilitators towards cognitive harmony.

The results of this study reiterate three key needs for future research. Firstly, cognitive labour must be understood as unique from other labour forms in order to reduce cognitive demand. Research similar to this study is needed to contribute to the visibility of cognitive labour and to new models. This will assist with its measurability and for cognitive labour to better addressed in future research as well as policies relating to family and paid work. Lastly, Future research needs to examine cognitive labour with different population groups as well as longitudinally to better understand it through the life course [19]. Our results highlight how differing contextual factors, including gender norms and couple dynamics influence how cognitive labour is performed and negotiated. This paper also supports the need to move beyond traditional theory and towards contemporary concepts, such as Geist’s adoption of the work-family fit model, where the needs of the entire family influence housework performance and to re-examine the methods we used to examine housework [81].

These findings support the use of a new lens for unpaid labour to promote gender equity, which, has not yet been achieved despite decades of research into housework as a concept [2, 11, 12]. These findings support this endeavour by providing couples with a new model through which cognitive labour can be viewed and more consciously divided. Interactions between each person formed four key themes: 1) habitually fostered patterns of trust; 2) agency in redefining family; 3) barriers to cognitive harmony; and 4) facilitators to cognitive harmony. Dyadic interviews allowed for the development of deep, couple-based narratives relating to cognitive labour performance. In line with research recommendations, this paper did not seek to explicitly compare cognitive labour performance between same-gender couples and heterosexual couples [53]. Instead, it provided a new lens through which it can be viewed outside of a traditional, heteronormative dynamic.

Moving forward, there needs to be a more nuanced understanding of housework performance outside of cisgender heterosexuality beyond an examination of time use and physical task allocation and division [81]. This can be achieved through a shift in the theoretical perspectives used and a focus on mixed methods studies. We have established the richness of data that can occur when using dyadic interviews to discuss cognitive labour and encourage its use to further explore household performance and the constructs behind it. The results of this study build on existing evidence of the need to shift the way we theorise and explore household labour as a construct and to challenge the existing status quo.
